# New discretization method applied to NBV problem: Semioctree

**DOI:** 10.1371/journal.pone.0206259

**Published:** 2018-11-01

**Authors:** L. M. González-deSantos, J. Martínez-Sánchez, H. González-Jorge, L. Díaz-Vilariño, B. Riveiro

**Affiliations:** 1 Applied Geotechnologies Group, Departament of Natural Resources and Environmental Engineering, University of Vigo, Vigo, Spain; 2 Fundación Centro Innovación Aeroespacial De Galicia, Nigrán, Spain; 3 Applied Geotechnologies Group, Departament of Materials Engineering, Applied Mechanics and Construction, School of Industrial Engineering, University of Vigo, Vigo, Spain; University of Science and Technology Beijing, CHINA

## Abstract

This paper presents a discretization methodology applied to the NBV (Next Best View) problem, which consists of determining the heuristical best position of the next scan. This new methodology is a hybrid process between a homogenous voxelization and an octree structure that preserves the advantages of both methods. An octree structure is not directly applicable to the NBV problem: as the point cloud grows with every successive scanning, the limits and position of the discretization, octree structure must coincide, in order to transfer the information from one scan to the next. This problem is solved by applying a first coarse voxelization, followed by the division of each voxel in an octree structure. In addition, a previous methodology for solving the NBV problem has been adapted to make use of this novel approach. Results show that the new method is three times faster than the homogenous voxelization for a maximum resolution of 0.2m. For this target resolution of 0.2m, the number of voxels/octants in the discretization is reduced approximately by a 400%, from 35.360 to 8.937 for the study case presented.

## 1. Introduction

The use of 3D information has increased greatly in recent years. Currently, there is a wide range of sensors that are used to obtain 3D information of the environment, such as cameras or laser scanners (LS). These sensors have increased their accuracy and decreased their price, so now the challenge is in the processing of the large amount of geometric information captured.

All this 3D information, in the form of a point cloud, can be used for the digital modelling of environments [1–4], such as historical buildings [5], or for indoor navigation of autonomous vehicles [6,7]. In this context, 3D environment modelling is gaining importance. Akinci *et al. [8]* use the 3D information obtained by a LiDAR sensor to perform construction quality control. Adán *et al. [9]* presents an algorithm that processes dense coloured 3D point clouds in order to recognize some small structural elements that are located on walls, such as sockets or extinguishers. To control structural elements in construction sector, the point cloud must achieve some accuracy and density requirements in order to reconstruct the scene correctly[10]. Valero et al. [11] uses LiDAR data for Cultural Heritage (CH) buildings surveying and maintenance, using a 2D Continuous Wavelet Transform (CWT) algorithm for the segmentation of the point cloud. 3D modelling combined with recent advances in robotics allows this process to be carried out autonomously using Unmanned Ground Vehicles (UGVs)[12] or Unmanned Aerial Vehicles (UAVs) [13]. Such vehicles are able to navigate autonomously and integrate sensors that capture 3D data of the environment. Several systems have already been developed for this purpose. The most widespread methodology is the use of SLAM (Simultaneous Localization and Mapping) algorithms [14] that are the foundation for the 3D data acquisition of the environment in previously unknown areas using LiDAR sensors. Another surveying method makes use of high resolution LS that must stay in statically during data collection, resulting in a slower scanning process [15]. For these methods, a series of scanning positions must be defined previously to obtain a complete point cloud. Therefore, the challenge for this method resides in the calculation of the minimum number of scanning positions that minimize occluded areas. This topic has been faced with in a large number of studies, known as the determination of Next Best Views (NBV) or Next Best Scan (NBS). Conolly *et al*. [16] presented a solution for the calculation of these NBVs early in 1985, in which the room is discretized using an octree structure, defining the visible, occupied and non-visible areas. Similar algorithms were used for 3D modelling of objects. Vasquez-Gomez *et al*. [17] presented a method based on discretizing the surface of the object and occlusions in voxels, followed by a visibility analysis to determine the next best scan position.

## 2. Related work

NBV positions gain special importance for indoors 3D modelling and, accordingly, several methods can be found in the literature that focused on its calculation. A Surface-Based NBV algorithm [18] consisted of finding the scan positions that minimize occlusions on the surface of the objects. This method was mostly used for object modelling [19]. A Volumetric-based approach algorithm [20] took into account the total volume of the room and discretized it into voxels. The NBV position was chosen to minimize occluded volumes. Other methods have been presented to solve the NBV problem. Surmann *et al*. [21] simplified the geometry of the room to a 2D map, in which the walls are represented by lines and the occluded areas as unseen lines, selecting as NBV the candidate that maximize the number of unseen-to-visible lines. Dierenbach *et al*. [22] used a hybrid method between a surface-based and a volumetric approach for the determination of the NBV for object 3D modelling, using both approaches sequentially.

This manuscript will focus on the volumetric approach methods, where numerous studies have already been carried out. Adan *et al*. [23] presented a method in which the room was discretized into voxels, which are small cubes of a defined size that represents the volume they contain, and labelled them as occupied, empty and occluded. NBV determination was carried out by a visibility analysis based on a ray-tracing algorithm. Prieto *et al*. [24] also classified the occupied voxels in structural elements and non-structural elements. Quintana *et al*. [25,26] presents a door location algorithm that use 3D coloured point clouds for door location in indoor environments. González-deSantos *et al*.[27] delimited the room by locating doors and windows, adding three new types of voxels: window, door and exterior. Quintana *et al*. [28] presents a NBV algorithm that locates the structural elements of buildings and use this as a RoI (Region of Interest) for the NBV calculation, giving more importance to this areas in the NBV calculation.

The main problem for these methods is the resolution of the discretization, in other words, the voxel size. These studies divided the whole room using a homogenous voxel discretization. In this context, a resolution improvement by decreasing the voxel size, results in a cubic growth in the number of voxels to be processed. This situation increases processing time. A possible solution to solve this problem consisted of applying a discretization based on an octree structure [29–31], increasing the resolution only in the areas that need it. However, the use of an octree structure does not result into homogenous discretization, so, as the point cloud size grows, the structuration between consecutive scans may not be coincident, because the initial cube will grow, so each subdivision will not have the same limits as in previous scans. In the main, this means that structure information could not be transferred from previous scans to the next one without losing homogeneity.

The main novelty of this study is the introduction of a new homogenous discretization method that allows us to increase the resolution only in the areas that requires a higher resolution, such as occupied areas or the limit between visible and occluded areas. This new discretization method applied to the calculation of the NBV, which is a hybrid procedure between a simple voxelization and an octree structure. First, a simple coarse voxelization and classification is applied to the entire point cloud. Afterwards, an octree structure is computed for the occupied voxels, where a higher resolution is needed. In this way, a higher resolution is obtained but with a homogenous discretization that allows us to transfer the information from previous scans to the next one.

With this discretization method the processing time is reduced significantly, reducing the number of voxels needed for the discretization. Finally, in the results section, this discretization method is compared with the homogenous voxel discretization, applying the two discretization method to the same study case, comparing the results.

The manuscript is organized as follows. Section 2 describes the proposed methodology. Section 3 reports the results obtained from applying the methodology to a real case study composed of several rooms and a discussion of those results. Finally, Section 4 concludes this work.

## 2. Methodology

**Fig 1:**
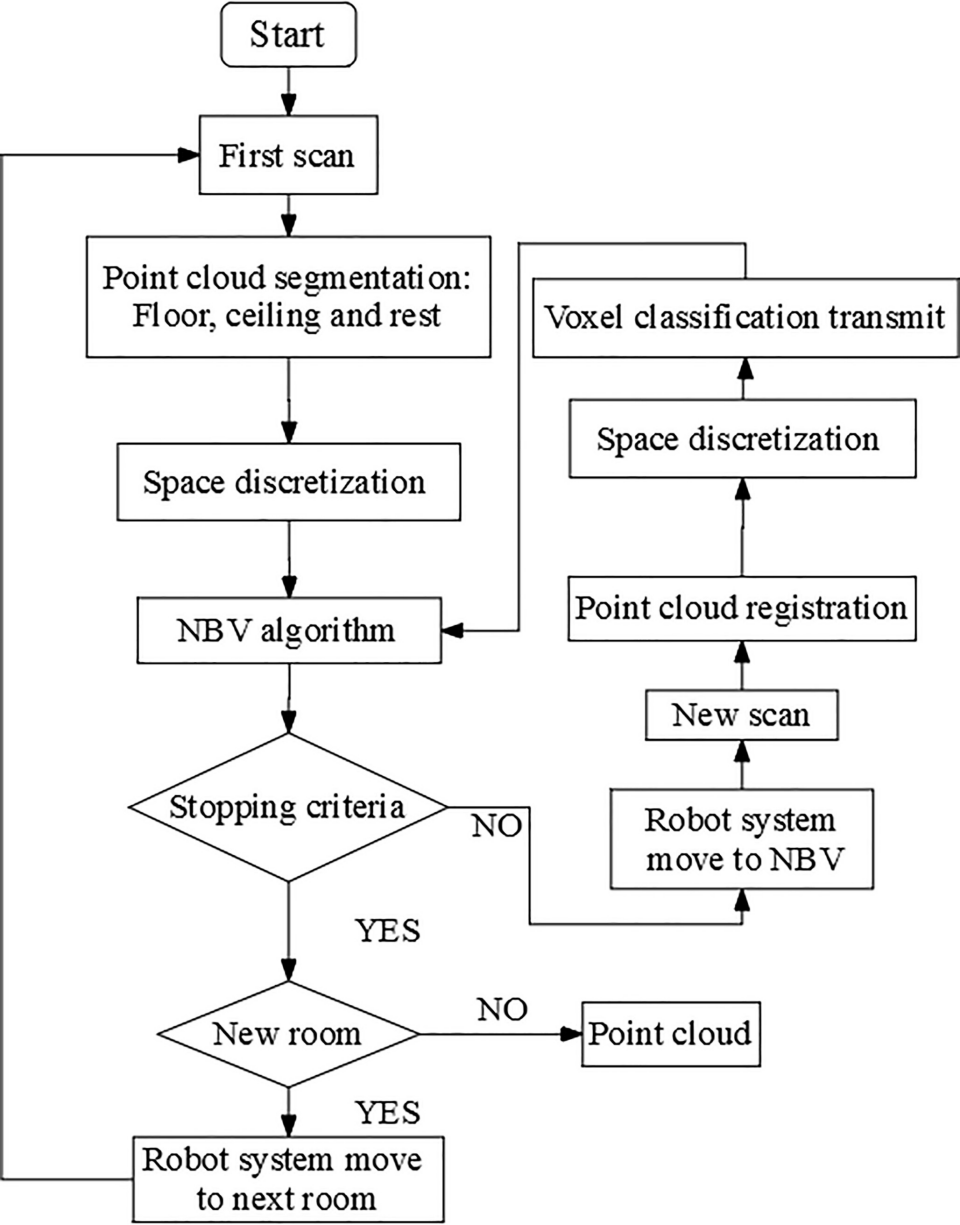
General workflow of the method.

The methodology can be summarized in three main steps, namely, point cloud pre-processing, point cloud structuration and NBV calculation. The general workflow is summarized in **Fig 1**. In this methodology, the use of the Robotnik Guardian [32] UGV has been simulated, taking into account the system features facilitated by the manufacturer. This UGV has a payload of 50kg and an autonomy of three hours. Similar systems have been previously used for this purpose [23,24,27]. This UGV makes use of SLAM algorithms for autonomous navigation, thus this manuscript will focus on NBV calculation. Final NBV positions will be transmitted to the UGV controller. Prior information of the room geometry is not used for NBV calculation, that lies only on the point clouds obtained with the LiDAR sensor.

### 2.1 Point cloud segmentation: Floor, ceiling and rest

As stated above, the only information used for the calculation of the NBV are the point clouds obtained with the LiDAR sensor carried by the UGV. All point clouds must be pre-processed before their analysis. The pre-processing consists of levelling the ceiling’s plane and making the floor’s plane coincident with the XY-plane, which means that the floor’s plane is at height zero. To this purpose, the ceiling/floor’s plane is calculated using a RANSAC (Random Sample Consensus) algorithm [33], where *n* = (*n*_*x*_,*n*_*y*_,*n*_*z*_) is the normal vector each plane, so the angles between the normal vector and the Z-axis are calculated using **Eq 1**, where α is its angle with the xz-plane and β is its angle with the yz-plane. These angles are used for point cloud rotation around X/Y-axis by applying a rigid body transformation (**Eq 2**). Finally, a translation by the floor’s plane height, is applied to make it coincident with the XY-plane.

$$\begin{array}{c} \alpha =atan\;\left(\frac{n_{x}}{n_{z}}\right)\\ \beta =atan\;\left(\frac{n_{y}}{n_{z}}\right) \end{array}$$ (1)

$$\begin{array}{c} Rot(x,\beta )=\left(\begin{array}{ccc} 1 & 0 & 0\\ 0 & cos(\beta ) & -sin(\beta )\\ 0 & sin(\beta ) & cos(\beta ) \end{array}\right)\\ Rot(y,\alpha )=\left(\begin{array}{ccc} cos(\alpha ) & 0 & sin(\alpha )\\ 0 & 1 & 0\\ -sin(\alpha ) & 0 & cos(\alpha ) \end{array}\right) \end{array}$$ (2)

**Fig 2:**
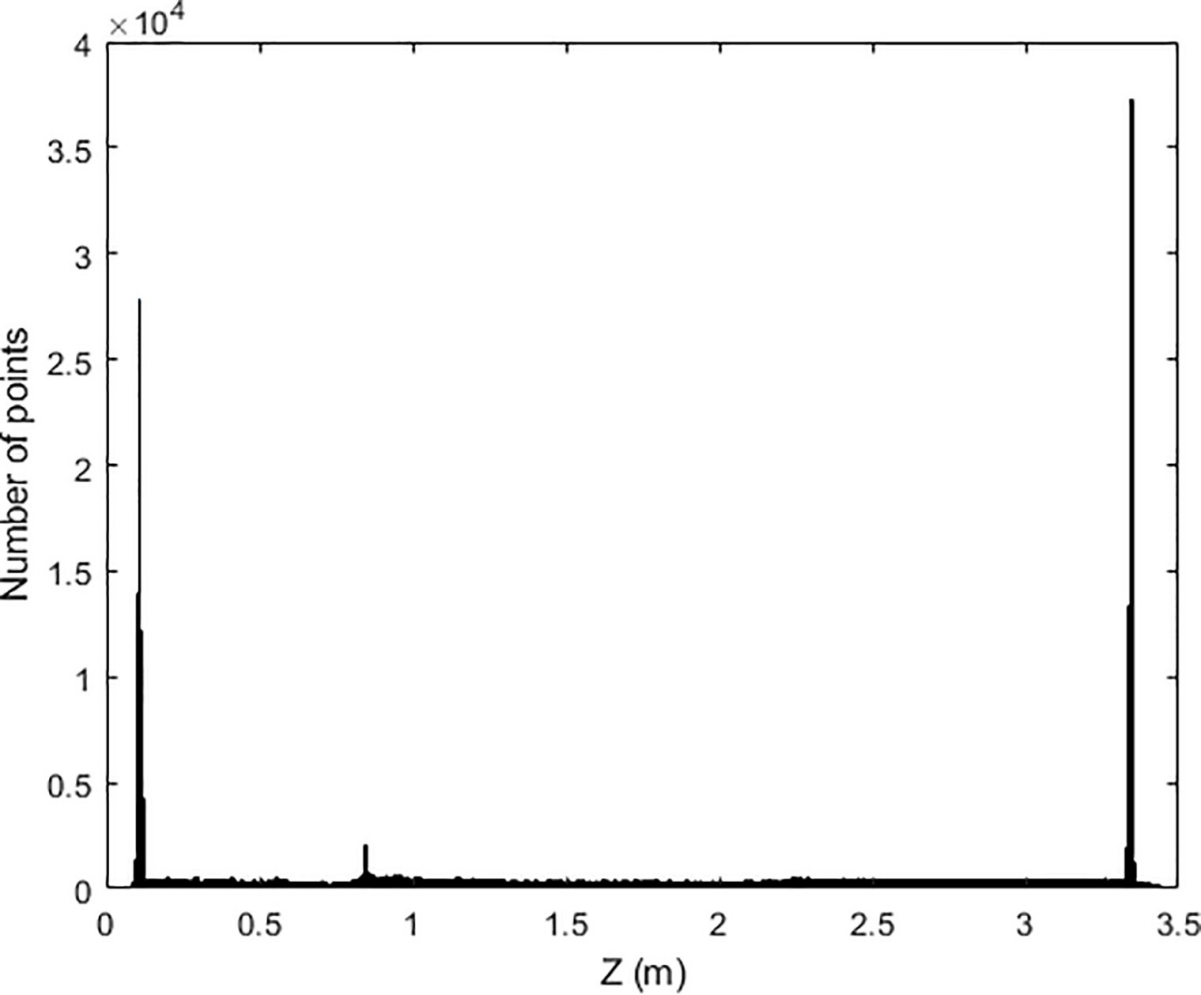
Z-histogram: Histogram of the elevations of the points. The maximum values correspond to the floor and ceiling of the room.

**Fig 3:**
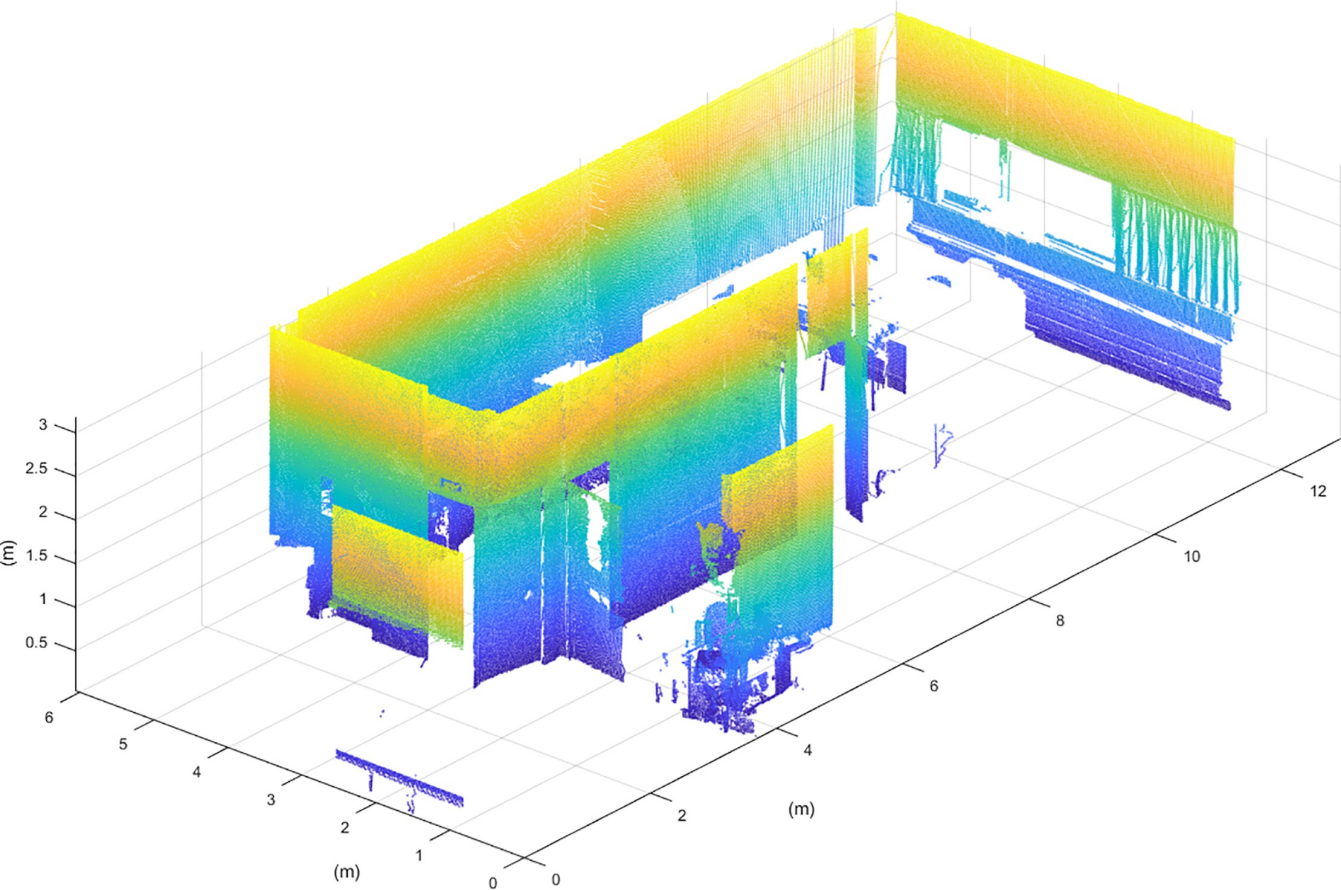
Segmented point cloud.

Once the point cloud is pre-processed, it is segmented in three regions, filtering the points belonging to the floor and the ceiling from the rest of the room. Segmentation is carried out by Z-coordinate histogram analysis (**Fig 2**). A Gaussian distribution is fitted to the peaks of the histogram corresponding to the heights of floor and ceiling, and its standard deviation, sigma, is derived. Therefore, a two-sigma threshold is used for the segmentation, so the 95% of the points belonging to the ceiling and floor are filtered (**Fig 3**). Since the floor and ceiling do not provide any information for NBV calculation, the segmented point cloud discretization is more manageable.

### 2.2. Point cloud discretization and classification

**Fig 4:**
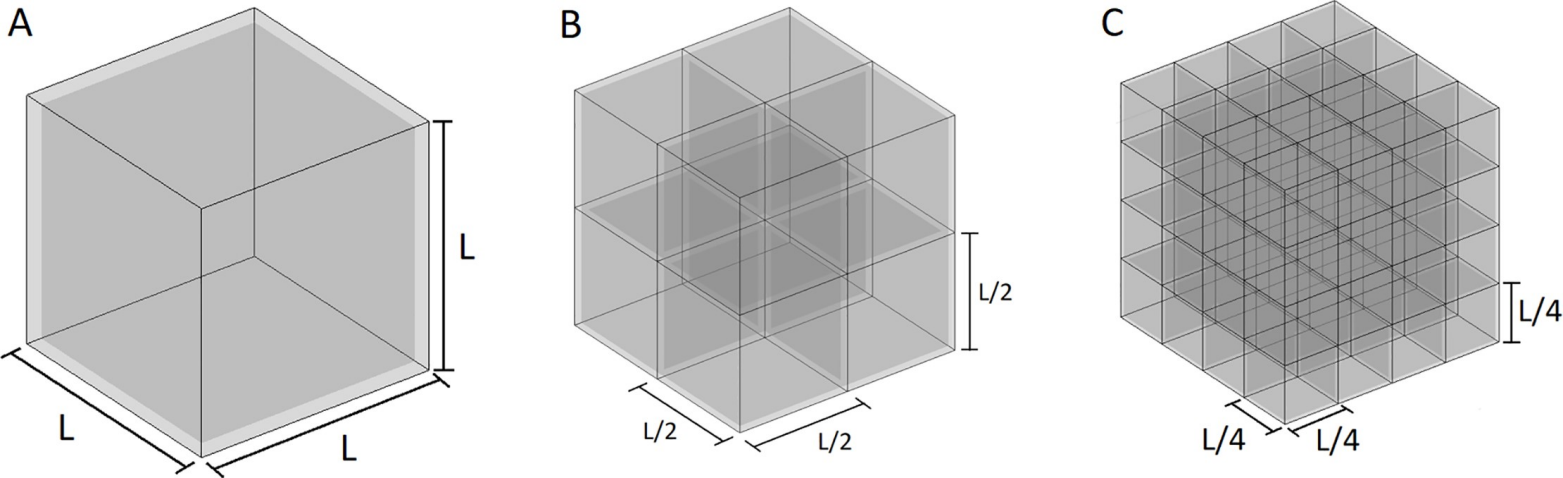
Three levels discretization. A: First level: voxel side = L. B: Second level: voxel side = L/2. C: Third level: voxel side = L/4.

After pre-processing, the point cloud undergoes a first coarse voxelization, using a similar method to that presented by Prieto *et al*. [24]. Then, an octree structuration is applied to the voxels that need a higher resolution, such as occupied voxels and those voxels in the limit between occluded and non-occluded areas. The authors have named this method of discretization as semioctree, because it is a hybrid procedure between a homogenous voxelization and an octree, that is obtained as in Conolly *et al*.[16]. For this purpose, the discretization is arranged in a matrix of voxels, in which each voxel has an octree structure. The octree structure consists of dividing a voxel into eight octants that can be recursively divided again into octants until reaching a maximum tree level, a defined minimum leaf size or until the structuration does not provide a higher resolution, as will be explained later. Let us consider the octree structure is divided in levels: **Fig 4** serves as an example and shows a structuration divided in three levels.

From here on, we will call octants the resulting cubes of each division of the levels of the octree structure, to differentiate them from the voxels of the first part of the discretization method.

The number of levels can be calculated taking into account the voxel size of the first level and last levels. The voxel size of each level is half the size of its parent.

This octree structure undergoes a classification, that considers six different classes:

- ‘Empty’: Empty voxel (which does not contain points) with a direct Line of Sight (LOS) from the scanner position.
- ‘Occupied’: Voxels that contains points.
- ‘Occluded’: Empty voxel without direct LOS from the scanner position.
- ‘Window’: Empty voxel that belongs to a window in the room.
- ‘Door’: Empty voxel that belongs to a door in the room.
- ‘Out’: Empty voxel with direct LOS through a window or door.
- ‘NBV’: a unique empty voxel that is the optimum next best scanning position.

This voxel classification is carried out in three steps: 1.-Occupancy analysis, 2.-location of doors and windows, and, 3.-occlusion analysis.

#### 2.2.1. Occupancy analysis

**Fig 5:**
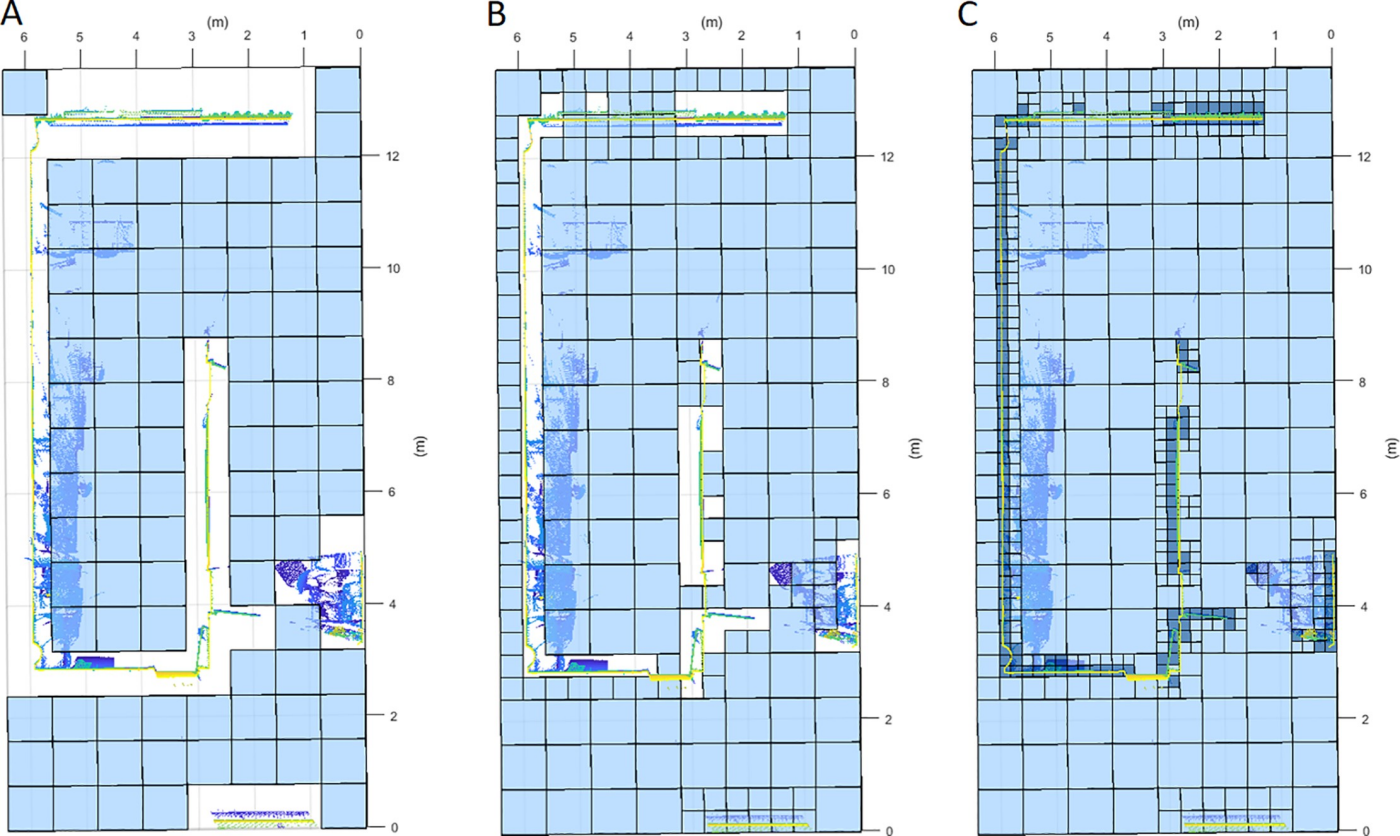
Horizontal discretization plane at the scanner height. Occupied (dark blue) and empty (light blue) voxel/octant clasification. A: First Level. Voxel side = 0.8 m. B: Second level. Voxel side = 0.4 m. C: Third level. Voxel side = 0.2m.

**Fig 6:**
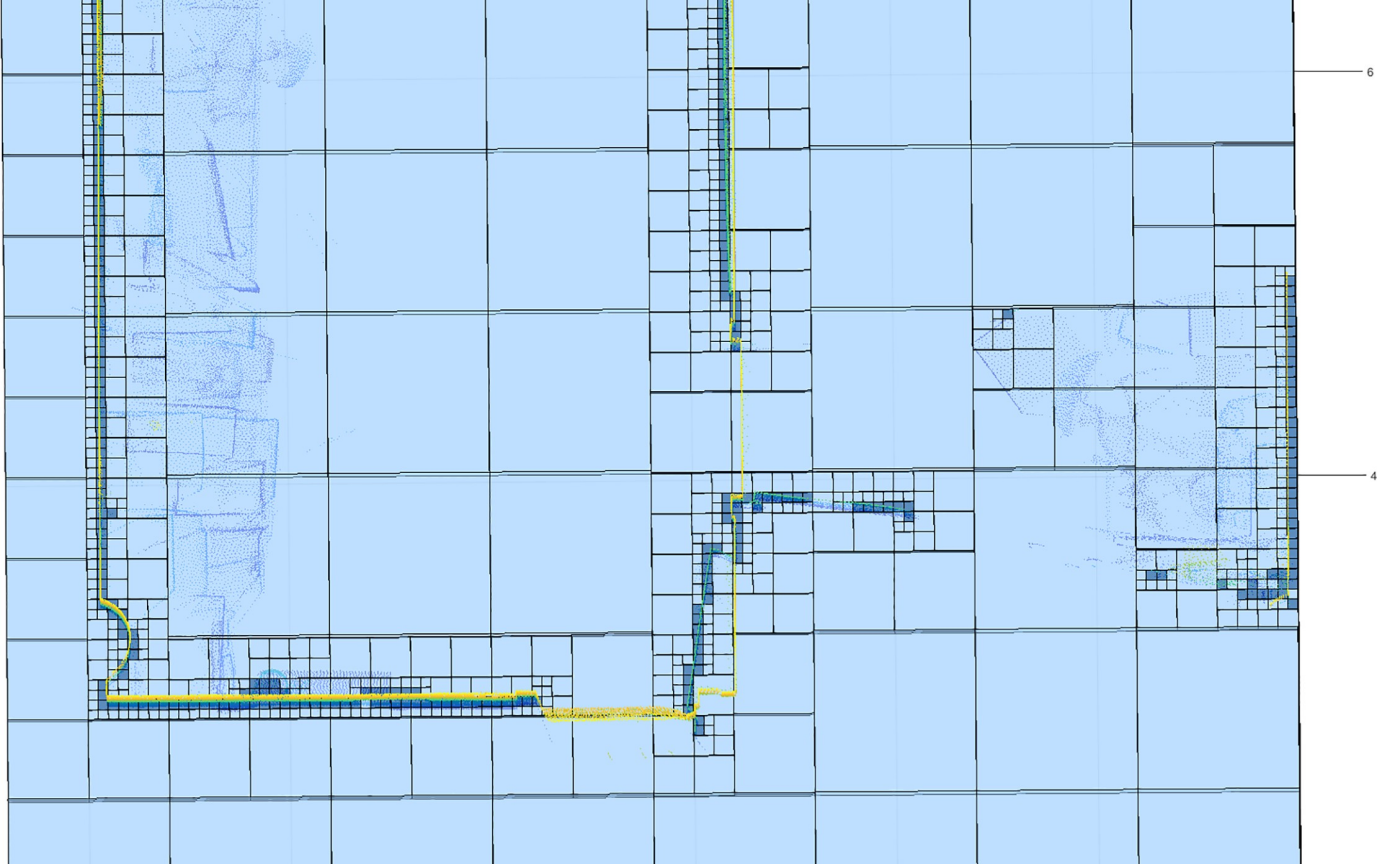
Horizontal discretization plane at the scanner height. Occupied (dark blue) and empty (light blue) voxel clasification. Occupancy study accuracy. Voxels size from 0.8 m to 0.05 m.

The occupancy analysis consists of classifying the voxels of the room in occupied and empty. The occupied areas of the room are divided in this step, obtaining an octree structure for each voxel in order to improve the resolution of these areas. The structuration is based on the recursive inspection of each level and subdivision of occupied leafs (divisions that contain points), as shown in **Fig 5**. As a result, the octree presents occupied voxels/octants only in the last level, so we can ensure a good resolution without needing to apply this voxel size to the entire discretization (**Fig 6**).

#### 2.2.2. Door and window location

For the location of doors and windows, each of the walls of the room is studied individually, extracting the points belonging to them using a RANSAC algorithm [33]. The wall is discretized using a grid of empty or occupied cells depending on the number of points inside each of them. This grid is treated like an image, using a pattern to look for rectangular holes that may be a door/window, using the method presented by González-de Santos *et al*. [27]. For this study case only rectangular windows are considered, for other types of windows, such as circular or oval windows it would be necessary to define different patterns. A visibility study is afterwards applied to the door/window candidates. The visibility study consists of a ray-tracing from the scanner position to the centre of the cells marked as a door/window. We consider a direct-LOS cell if the ray path does not intersect any occupied voxel. If the ray path crosses trough occupied voxels it means that there is not a direct LOS to the candidate.

**Fig 7:**
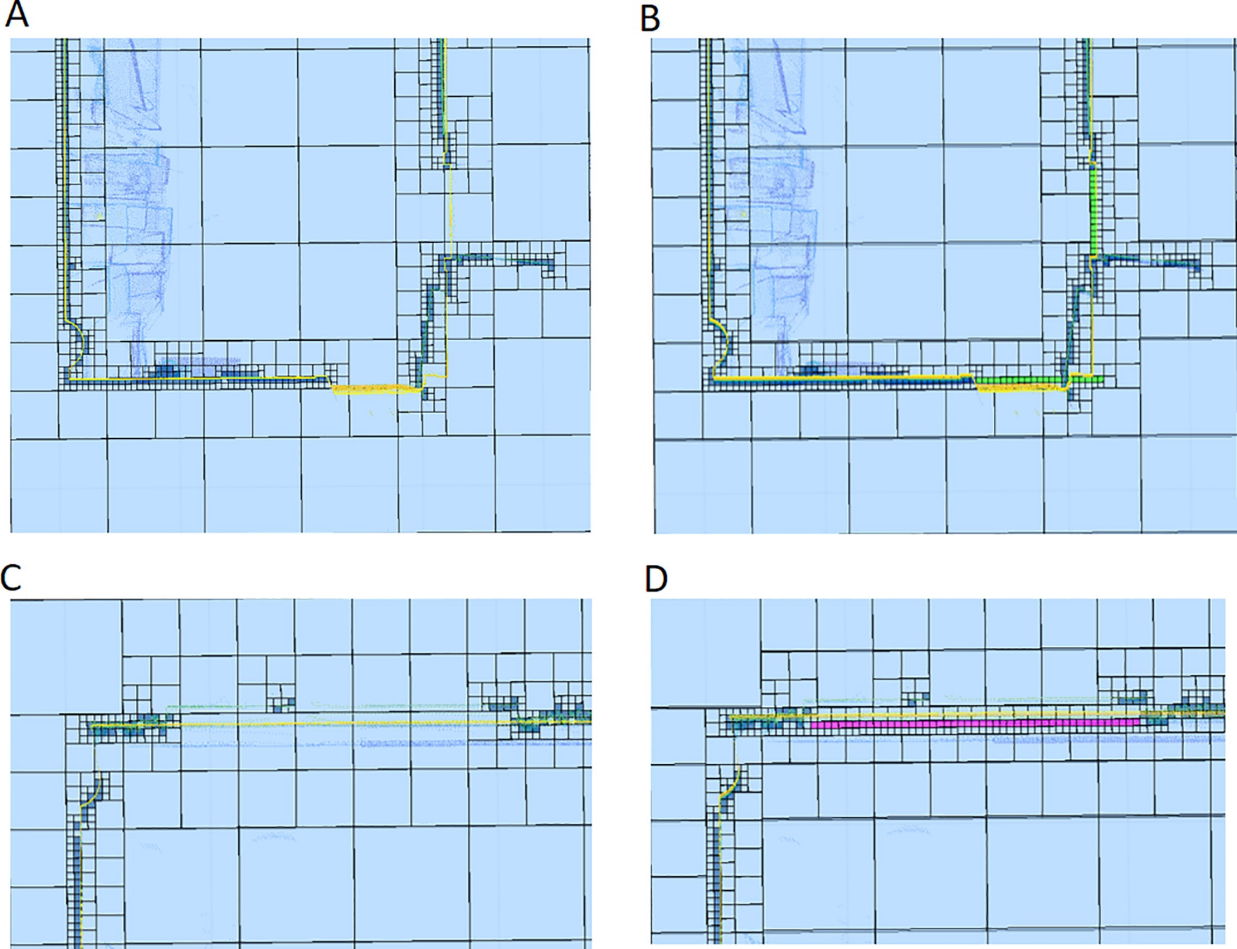
Horizontal discretization plane at the scanner height. Door and window location. Light blue: empty; Dark blue: occupied; Green: door; Magenta: window. A: Discretization before door location. B: Discretization after door location. C: Discretization before window location. D: Discretization after window location.

If the percentage of direct-LOS cells in the candidate is below 85% the pattern is marked as a false positive that may be produced by a rectangular-shaped occlusion and discarded. If the candidate is confirmed, the door/window label is transferred to the octants in the last level of the octree (**Fig 7**).

#### 2.2.3. Occlusion study

Once the ‘empty’ and ‘occupied’ octants are classified and the door and windows are located, an occlusion study is carried out to precise the labelling of empty octants/voxels. The remaining classes that we must consider in this stage are the following: areas not visible from the scanner position (‘occluded’) and outdoor (‘out’) voxels. The occlusion analysis is based on a ray tracing algorithm using a method similar to Adán *et al*. [23]. At this point, if the ray path to the target crosses an occupied octant, the target octant is labelled as ‘occluded’. If the ray path passes through a door/window octant and not occupied octants, the target is labelled as ‘out’.

**Fig 8:**
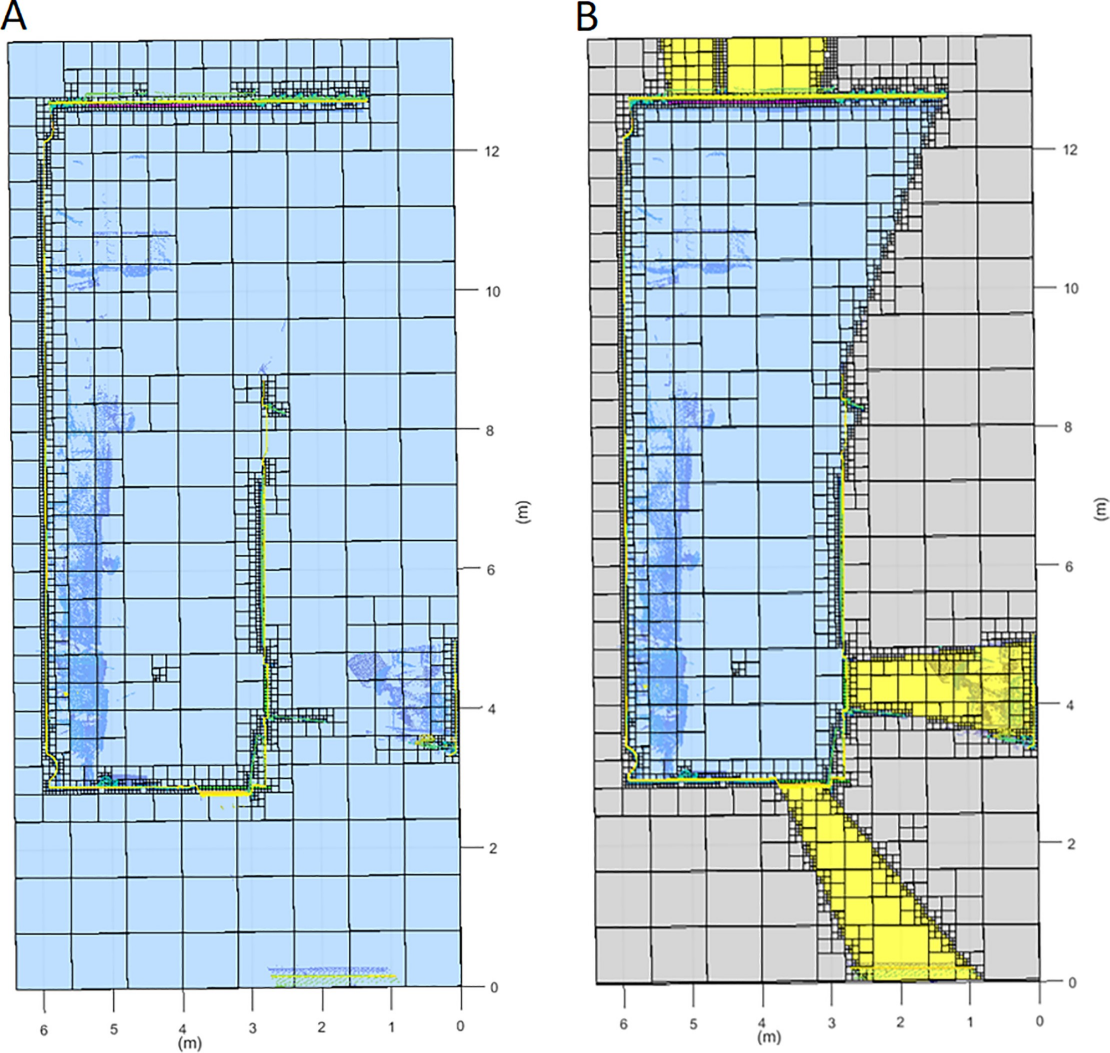
Horizontal discretization plane at the scanner height. Light blue: empty voxel/octant; Dark blue: occupied voxel/octant; Grey: occluded voxel/octant; Yellow: out voxel/octant; Black: Scan position. A: Discretization before the occlusion study. B: Discretization after the occlusion study.

In order to achieve a better resolution in the boundary between occluded and visible areas, this study is carried out in the last level of the octree structure, taking into account the values of the previous level subdivision and dividing the voxels/octants that are not completely occluded (**Fig 8**).

### 2.2. NBV calculation

The NBV calculation method presented by González-de Santos *et al*. [27] is adapted to the structuration presented in this manuscript. This NBV calculation method consists of verifying the feasibility as NBV candidates of the octants belonging to the horizontal plane at the height of the LS. For an octant to be a candidate, there must not be occupied or occluded voxels/octants within a safety cylinder from the floor to the top of the LS. All octants that meet the security criteria undergo a visibility analysis to check how many occluded voxels have direct LOS from the NBV candidate. The NVB is the candidate with a direct-LOS to the maximum number of occluded voxels/octants.

In this case, since the octree structure has several levels of octants of different sizes, two methods have been considered for the calculation of the NBV. The first method consists of applying the NBV calculation to all the levels of the discretization and weight the result by the size (tree-level) of the occluded voxels/octants with direct LOS from the candidate. Therefore, an octant scores 8 times more than its children and the eight part of its parent. This method is faster but over-scores low resolution occluded areas. The second method consist of selecting a tree level to carry out this NBV calculation, taking into account the information from the rest of the levels. In this case, the resolution is considered uniform throughout the discretization. As a drawback, the smaller the octant size of the selected level, the longer the processing time of the method. To select the NBV octant, each NBV candidate start with a score *S*_*nCandidate*_ equal to zero, to which a *S*_*i*_ score is added for each occluded octant (**Eq 3**).

$$S_{nCandidate}=\sum_{i=1}^{nOccludedVoxels}S_{i}$$ (3)

The score *S*_*i*_ of each occluded octant studied can have three values according to the result of the visibility study. We have direct LOS (Line of Sight) of the studied occluded octant when the line of sight from the scanning octant to the study octant does not cross any occupied or occluded octant. If this line of sight crosses occluded octants but not occupied octants we have semi-direct LOS. If the line of sight crosses a occupied octant, we have no-direct LOS.

- Zero-point score if there was a no-direct LOS of the study octant from the scanner position.
- One-point score if there was a direct LOS of the study octant from the scanner position.
- In case of semi-direct LOS, the score is calculated according to the **Eq** (4), where *n*_*CrossedO*_ is the

number of occluded octants crossed.

$$S_{i}=\frac{1}{{1+n}_{CrossedO}}$$ (4)

**Fig 9:**
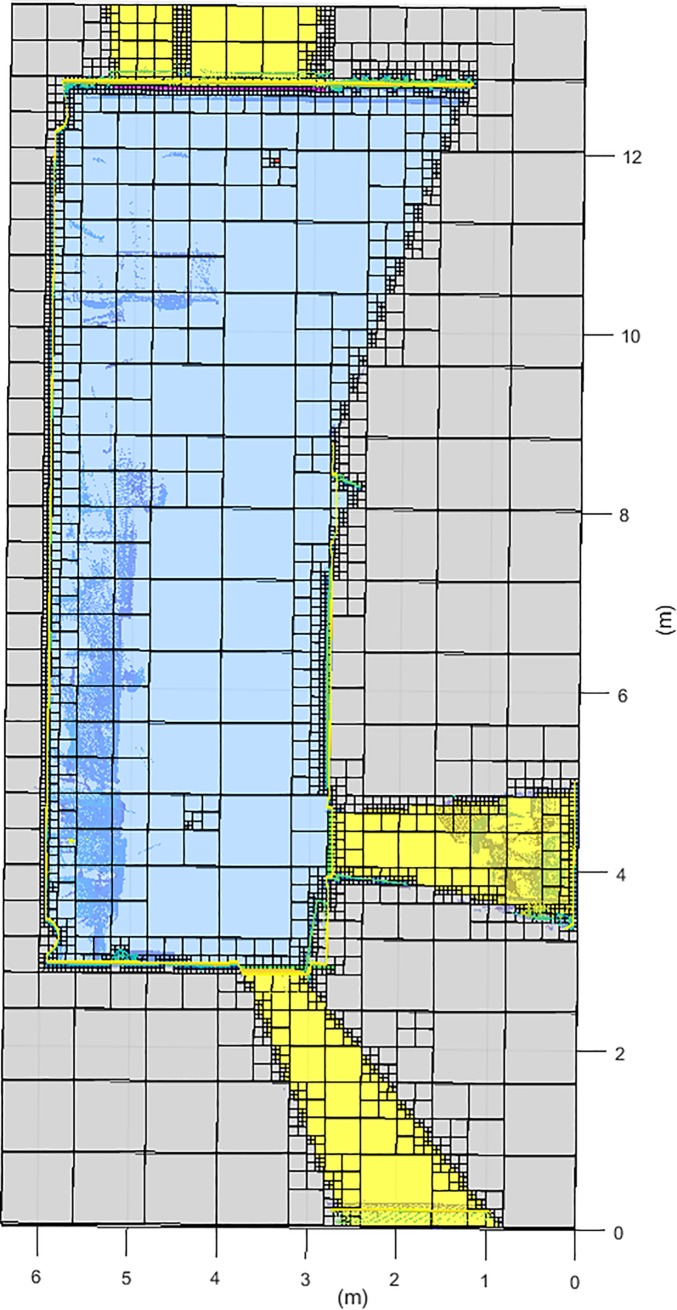
Horizontal discretization plane at the scanner height. **NBV calculated in a discretization from 0.8 m to 0.05 m**. Dark blue: occupied voxel/octant; Light blue: empty voxel/octant; Yellow: out voxel/octant; Green: door voxel/octant; Magenta: window voxel/octant; Grey: occluded voxel/octant; Black: scan voxel/octant; Red: NBV.

For our case study, we select the last level of the octree structure, to have the highest possible resolution (**Fig 9**).

### 2.3. Data transfer between successive scans

**Fig 10:**
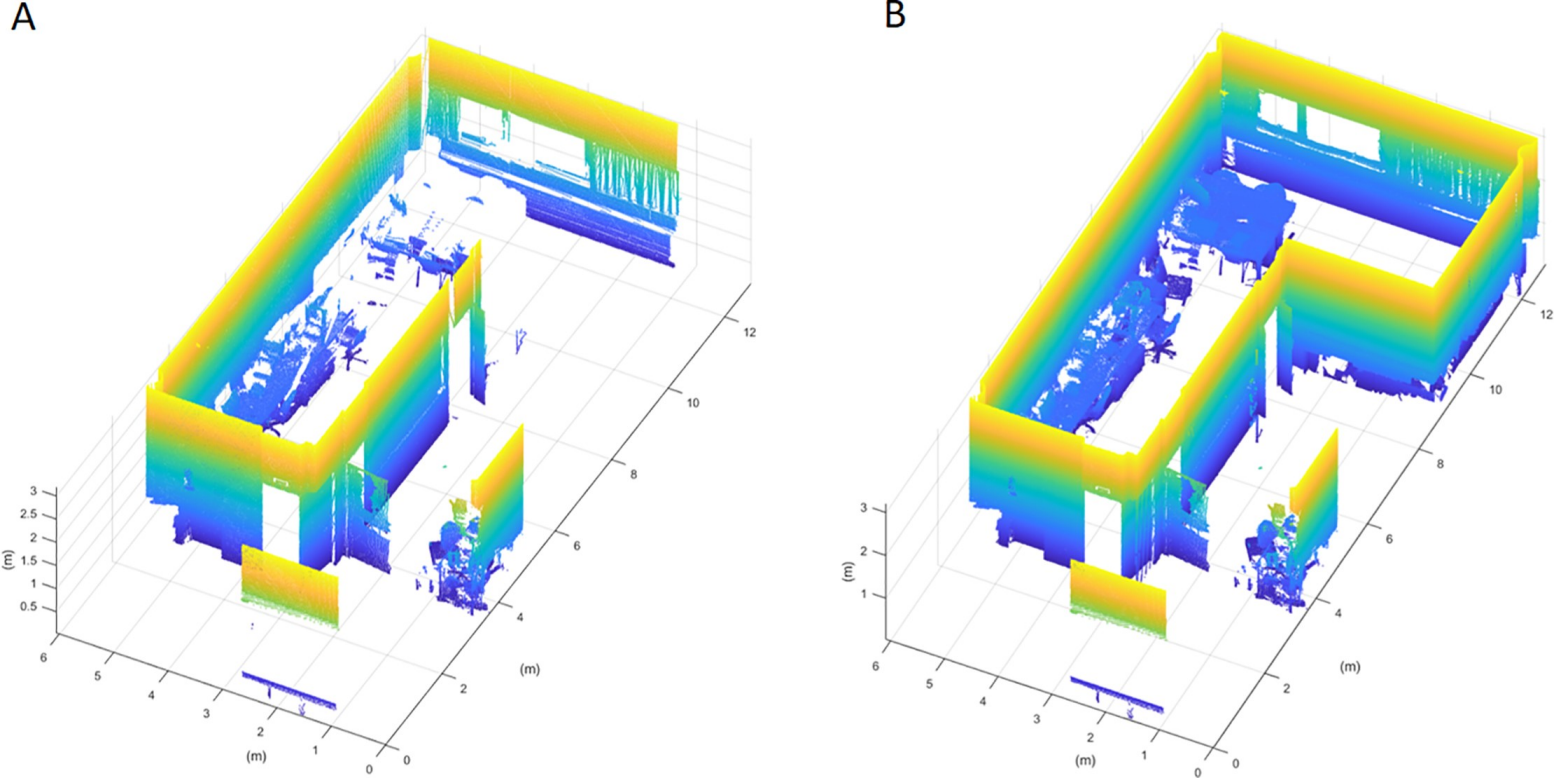
Horizontal discretization plane at the scanner height. Point cloud registration of successive scans. A: First scan point cloud. B: Second scan registered with the first one.

After NBV calculation, the robot moves to this position and starts a new scanning. Once the scan is finished, the point cloud is pre-processed to filter the floor and the ceiling. Floor and ceiling are used to register the new scan position to the previous scans. The registration is performed in a two-step fashion with a first coarse registration based on the robot navigation followed by a fine registration by an Iterative Closest Point (ICP) algorithm (**Fig 10**), as used by Prieto *et al*. [24].

**Fig 11:**
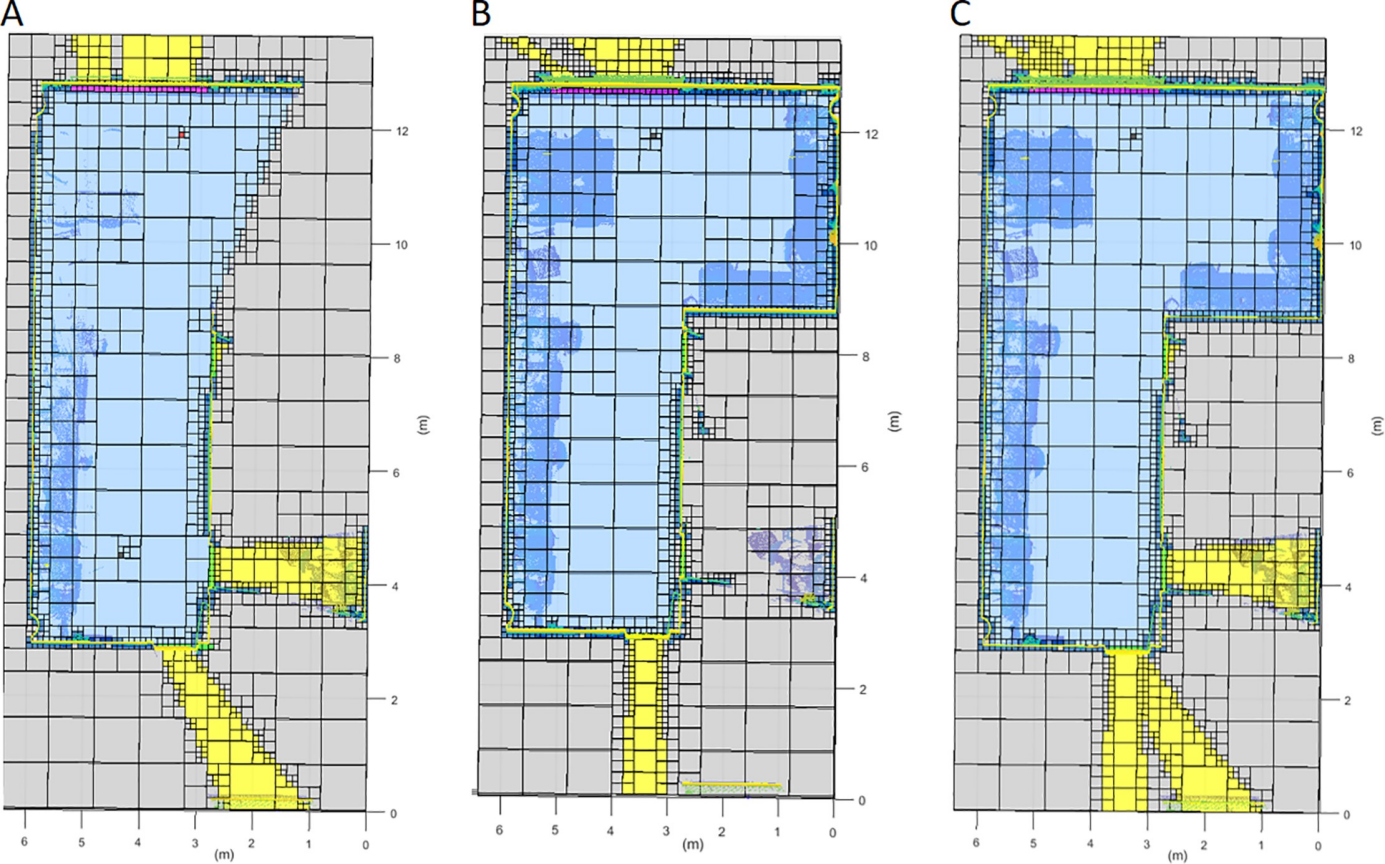
Horizontal discretization plane at the scanner height. Data transfer between successive scans. Dark blue: occupied voxel; Light blue: empty voxel; Yellow: out voxel; Green: door voxel; Magenta: window voxel; Grey: occluded voxel; Black: scan voxel; Red: NBV. A: First scan discretization. B: Second scan discretization. C: Resulting discretization.

The registered point cloud is merged with the previous dataset. The complete point cloud undergoes a new discretization and classification, as described in the previous sections, but taking into account the previous labelling (**Fig 11**). At this point, the information with a higher resolution and direct vision from the new scan position is prioritized in case of inconsistency.

The surveying stopping criteria consists of calculating the percentage of the total volume represented by the occluded voxels which are visible from the NBV, using **Eq 5,** where *vol*_*Voccluded*_ represents the volume of occluded voxels that are visible from the NBV, *vol*_*Voccupied*_ represents the volume of occupied voxels of the room, *vol*_*Vempty*_ represents the volume of empty voxels of the room and *vol*_*Vout*_ the volume of out voxels of the room. The lower the value of the Stop Percentage used, the more complete the resulting point cloud. A zero-value for stop percentage implies that the room has been scanned completely.

$$StopPercentage=\left(\frac{{vol}_{Voccluded}}{{vol}_{Voccluded}+{vol}_{Vempty}+{vol}_{Voccupied}+{vol}_{Vout}}\right)\;x\;100$$ (5)

## 3. Results and discussion

**Fig 12:**
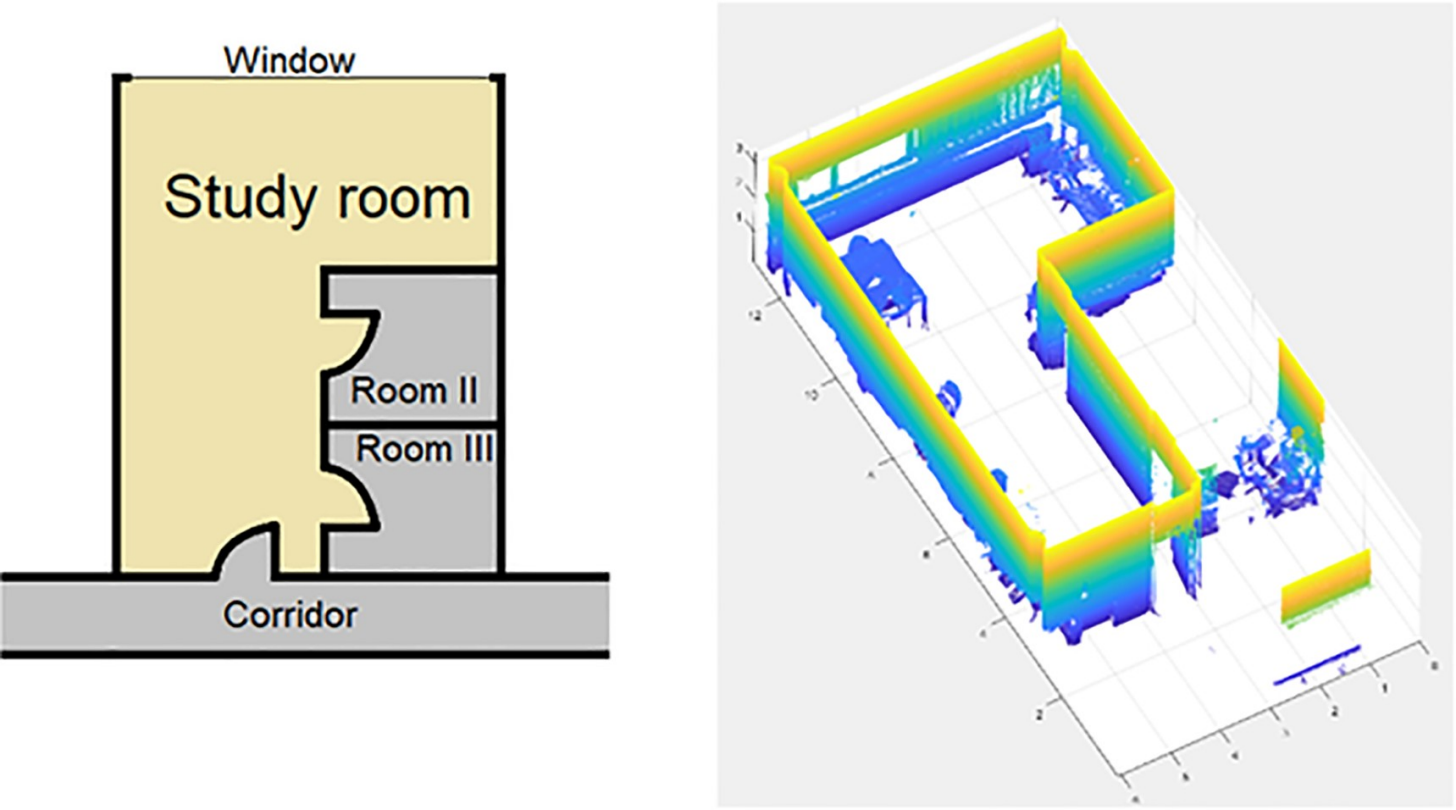
Sketch and point cloud of the study room.

This study was carried out in a laboratory of the School of Mining and Energy Engineering at the University of Vigo, and the results obtained are compared with the presented discretization method and a homogenous voxelization. This laboratory is composed of a main room with a window and three doors, that give onto two small rooms and a corridor (**Fig 12**). Three scans were needed to achieve a Stop Percentage of 0.1% with the presented discretization method. Each scan contains 20.4 million points, that were decimated with a distance filter, remaining a total of 400.000 point per scan. The methodology has been implemented in MatLAB R2017b with the Point Cloud Toolbox, installed on a computer with an Intel i7 processor and 16GB of RAM.

**Fig 13:**
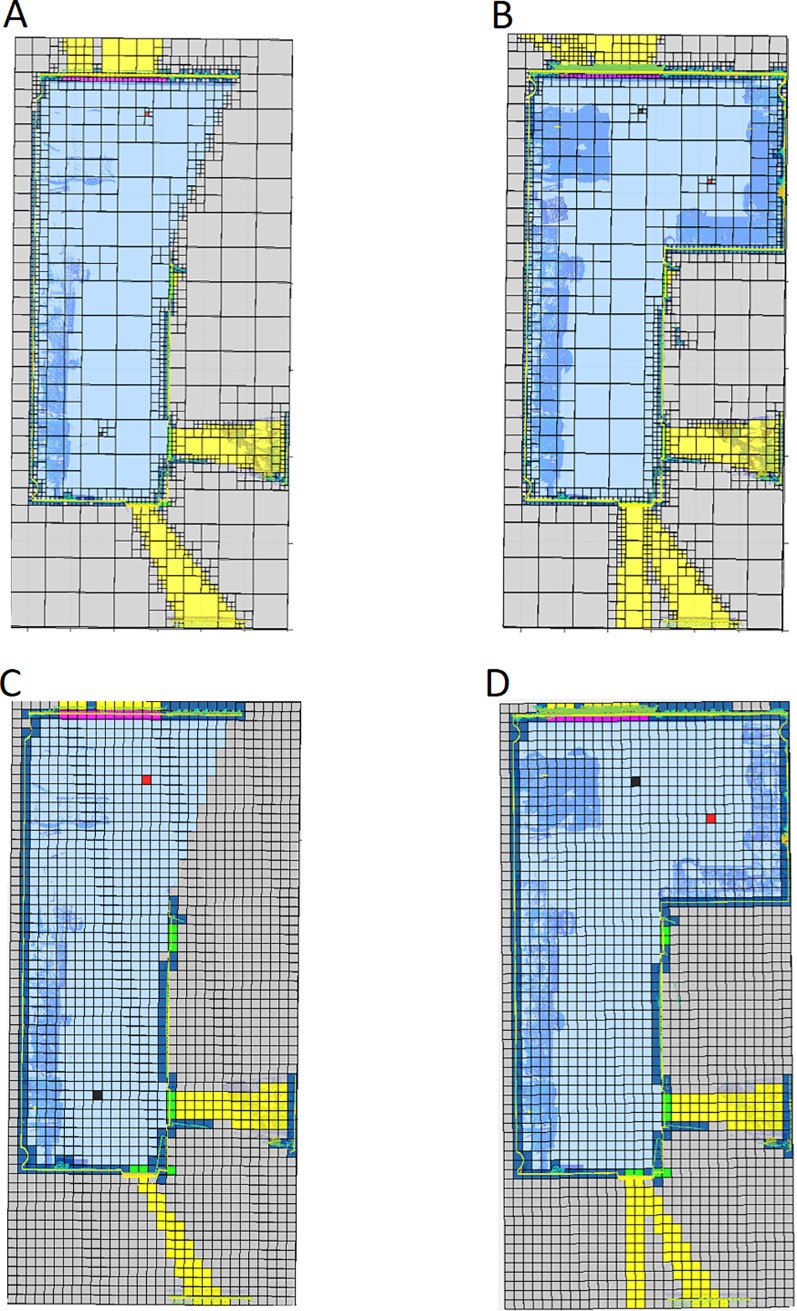
Horizontal discretization plane at the scanner height. **Comparison between the discretization presented in this manuscript (voxel size from 0.8m to 0.1m) and a discretization based on a homogenous voxelization (voxels size = 0.2m)**. Dark blue: occupied voxel/octant; Light blue: empty voxel/octant; Yellow: out voxel/octant; Green: door voxel/octant; Magenta: window voxel/octant; Grey: occluded voxel/octant; Black: scan voxel/octant; Red: NBV. A: First scan, new discretization. B: Second scan, new discretization. C:First scan, simple voxelization. D:Second scan, simple voxelization.

**Fig 14:**
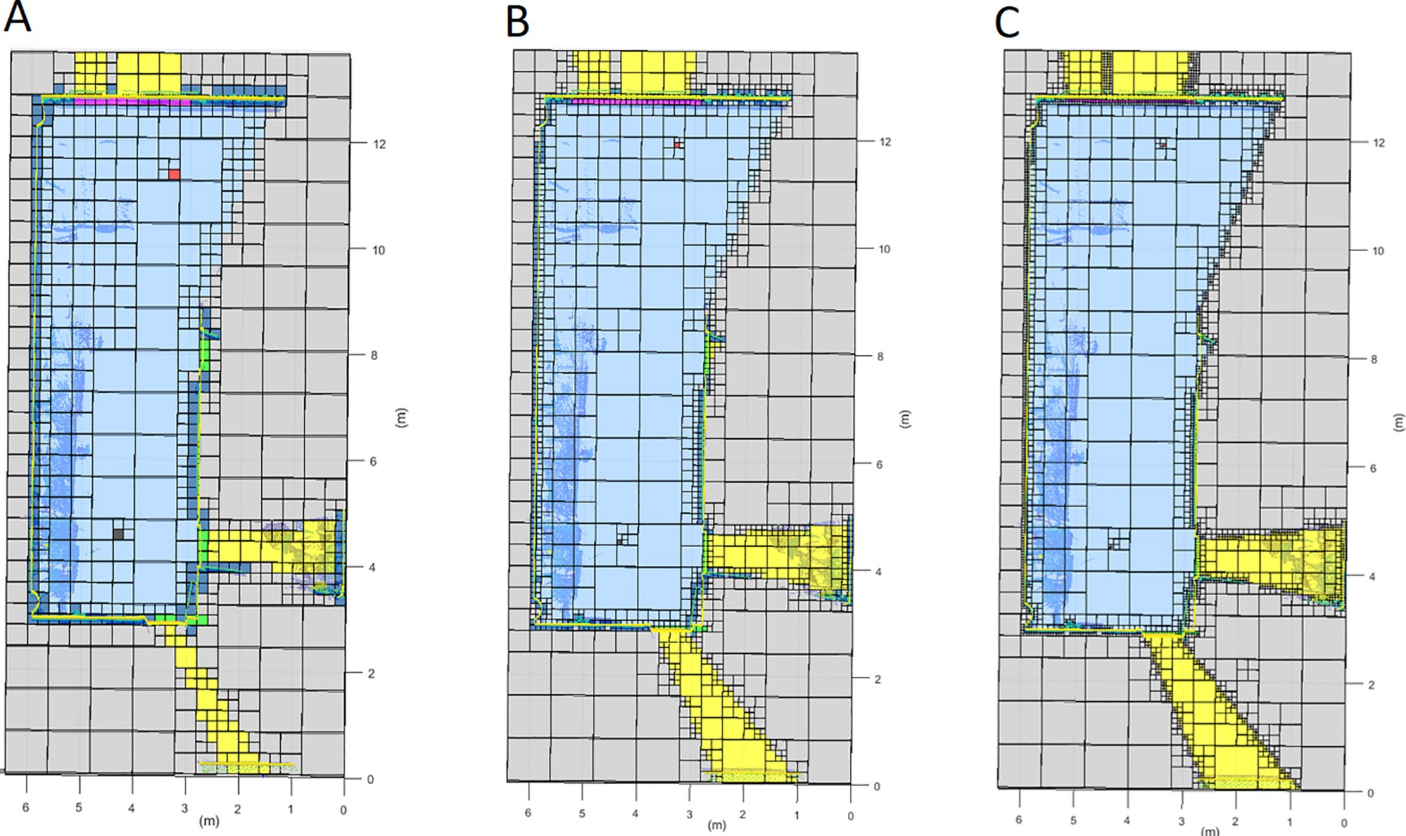
Different minimum voxel/octant size for the point cloud discretization. Dark blue: occupied voxel/octant; Light blue: empty voxel/octant; Yellow: out voxel/octant; Green: door voxel/octant; Magenta: window voxel/octant; Grey: occluded voxel/octant; Black: scan voxel/octant; Red: NBV. A: Voxel size from 0.8m to 0.2m. B: Voxel size from 0.8m to 0.1m. C: Voxel size from 0.8m to 0.05m.

Regarding the discretization, **Fig 13** shows the comparison between the new method presented in this manuscript and a homogenous voxelization. As can be seen, the new discretization shows more resolution (lower voxel size) in the occupied areas and less resolution (higher voxel size) in the areas with a lower number of points where a high resolution is not necessary. The voxel size depends on the size of the first and last levels of the discretization, that are fixed by the user (**Fig 14**).

**Fig 15:**
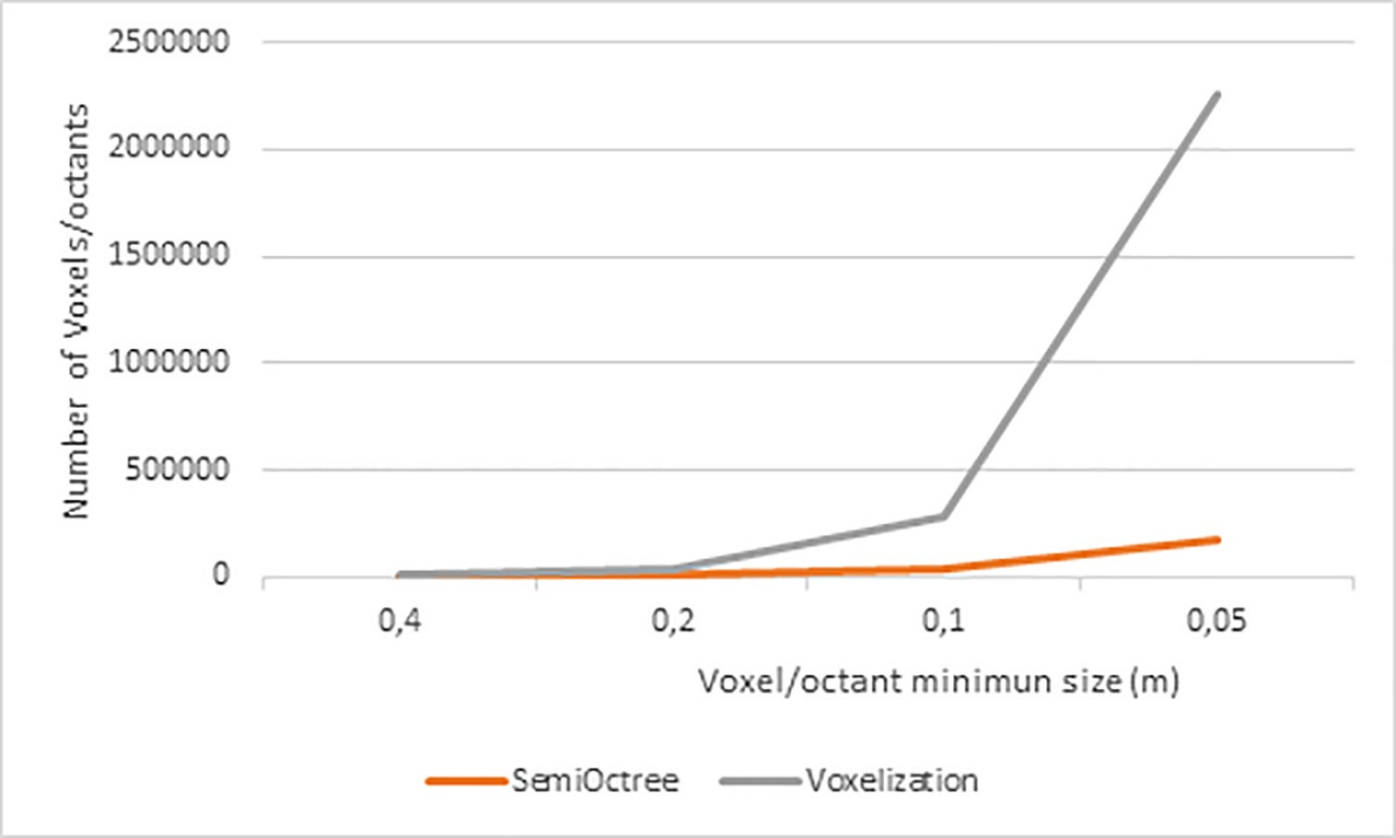
Comparison between the number of voxels/octants used to achieve a resolution with a homogenous voxelization and the presented method.

With this new discretization method, the processing time has been reduced. For a maximum resolution of 0.2m, the processing time has been reduced by 300%, from 1 minute 30 seconds to 30 seconds. This is due to the much lower number of voxels/octants needed to achieve a specific resolution with the semioctree presented in this paper, as shown in **Fig 15**.

**Table 1** shows a comparison between the volumes of each type of voxel in the voxelization and semioctree. As expected, the higher resolution used in the discretization, the percentage of voxels/octants used to define the occupied areas, doors and windows is lower.

**Table 1:** Percentage of the volume of voxels/octants of each class for each discretization.

	Voxel/octant size	Empty	Occupied	Occluded	Door	Window	Out
Voxelization	0.2m	55.69	7.07	34.16	0.31	0.14	2.67
Semioctree	0.8m to 0.2m	30.71	8.26	57.86	0.28	013	2.76
	0.8m to 0.1m	33.44	3.83	57.70	0.17	0.09	4.76
	0.8m to 0.05m	34.57	1.68	57.70	0.13	0.05	5.87

In **Fig 15** can be seen the growth in the number of voxels/octants needed to achieve a higher resolution. For a resolution target of 0.05m, with a homogenous voxelization 2.263.040 voxels are used for a homogenous voxelization of the study case, meanwhile 168.446 octants are used for a semioctree discretization of the study room, 13,4348 times less.

The increment factor is the ratio between the voxels/octants of a discretization and the voxels/octants needed to duplicate the resolution, such as from 0.4m to 0.2m of voxels/octants side. For the homogenous voxelization this increment factor is constant and equal to 8, while for the presented discretization method it is not constant, with an average value of 4,3243, as can been seen in **Table 2**.

**Table 2:** Increment factor for each resolution increase.

	From 0.4m to 0.2m	From 0.2m to 0.1m	From 0.1m to 0.05m	Average
Voxelization	8	8	8	8
Semioctree	4.2884	4.2544	4.4302	4.3244

## 4. Conclusions

This work presents the semioctree, a discretization method that allows improving the resolution without increasing the process load, focussing only on the critical zones. Such semioctree was afterwards applied to the NBV problem, improving the results obtained with the homogenous voxelization, which is the discretization method used in most of the studies that deal with the NBV topic. In terms of processing time to obtain a determinate resolution, an improvement by 300% was achieved for a maximum resolution of 0.2m. This result is based on the number of voxels/octants used in the discretization, that was reduced by 400% using the semioctree.

As future work, this method will be improved by defining ROIs (Region of Interest) in which the resolution will be even greater. This procedure which will allow NBV calculation to be applied to highly occluded areas, such as the pipe areas of a ship’s engine room or other industrial facilities.
